# Terrestrial solar radiation driven photodecomposition of ciprofloxacin in clinical wastewater applying mesostructured iron(III) oxide

**DOI:** 10.1007/s11356-020-10899-6

**Published:** 2020-09-29

**Authors:** Daniel R. Wagner, Kevin Ament, Lina Mayr, Thomas Martin, André Bloesser, Holger Schmalz, Roland Marschall, Friedrich E. Wagner, Josef Breu

**Affiliations:** 1 Bavarian Polymer Institute (BPI) and Department of Chemistry, University of Bayreuth, 95440 Bayreuth, Germany; 2 Physics-Department E15, Technical University of Munich, James-Franck-Straße, 85748 Garching, Germany

**Keywords:** Wastewater treatment, Photocatalysis, Ciprofloxacin, Fe_2_O_3_, Mesostructured, Cylindrical polymer brushes

## Abstract

Cationic cylindrical polymer brushes based on polybutadiene-*block*-poly(2-vinylpyridine) were applied as structure-directing agent for mesostructuring Fe_2_O_3_ nanoparticles into nanotubes. After temperature-controlled template removal, the obtained non-woven catalysts were tested for the photodegradation of ciprofloxacin under terrestrial solar radiation. At a slightly basic pH value, as typically encountered in clinical wastewaters, the mesostructured Fe_2_O_3_ shows a 4.5 times faster degradation of ciprofloxacin than commercial Aeroxide® TiO_2_ P25. Even wide-bandgap ZnO, mesostructured in the same way, is 1.6 times slower. Moreover, the non-woven-like structure of the catalyst allows for easy recovery of the catalyst and operation in a continuous flow reactor.

**Electronic supplementary material:**

The online version of this article (10.1007/s11356-020-10899-6) contains supplementary material, which is available to authorized users.

**Figure:**
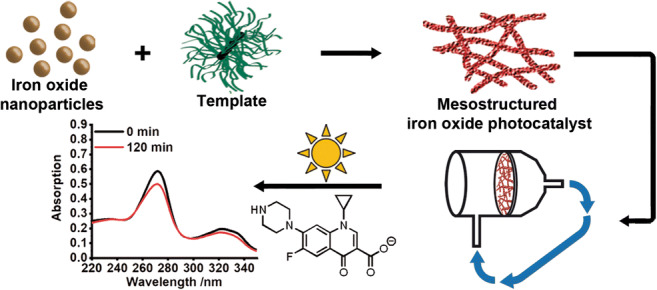
Graphical abstract

## Introduction

The removal of pharmaceutical residues like β-blockers, antihistamines, X-ray contrast media, or antibiotics from clinical wastewater represents a serious challenge because of their microbial toxicity (Bound and Voulvoulis 2005; Pal et al. 2010; Jiang et al. 2013; Loos et al. 2013). In Europe, ciprofloxacin is the most commonly ascribed fluoroquinolone antibiotic. As a broad spectrum antibiotic, active against gram-positive and gram-negative bacteria, it is applied against complicated bacterial infections of the urinal tract or skin infections, especially in hospitals (Martins et al. 2008; Van Doorslaer et al. 2011; Costa et al. 2018). For hospitals, a daily water consumption between 400 and 1200 L per bed is usual (Boillot et al. 2008). These clinical wastewaters contain up to 101 μg L^−1^ ciprofloxacin, and the level steadily increased by 15 % since the 1990s (Hartmann et al. 1998; Lindberg et al. 2004). Toxicity against prokaryotic and eukaryotic organisms hampers standard biological treatment of the wastewater (Brown et al. 2006).

Consequently, clinical wastewaters need pretreatment before entering municipal wastewater treatment plants. One promising approach is the in situ generation of highly reactive radical species via “advanced oxidation processes (AOPs).” In photocatalytic AOPs, these reactive species can be generated by photocatalytic transition metal oxides such as ZnO or the well-established Aeroxide® TiO_2_ P25 from Evonik. Utilizing solar energy as clean energy source to drive AOPs makes photodegradation most attractive (Gaya and Abdullah 2008; Paul et al. 2010; Khan et al. 2015; Guo et al. 2019).

In our previous work, we were able to show that ZnO nanotubes are superior over Aeroxide® TiO_2_ P25 in the photocatalytic degradation of ciprofloxacin (Bojer et al. 2017). Because of the slightly basic pH found for clinical wastewaters (pH ≈ 8), adsorption of ciprofloxacin, which at this pH is an anion (isoelectric point (IEP) at pH = 7.4) onto the positively charged surface of ZnO nanotubes (point of zero charge (PZC) at pH = 8.9), is electrostatically driven. Consequently, and contrary to ZnO, the like-charged surface of TiO_2_ (PZC = 6.5) hampers an efficient photocatalytic degradation. Unfortunately, the large bandgap of both TiO_2_ (3.2 eV (Wu et al. 2015)) and ZnO (3.4 eV (Bojer et al. 2017)) allow only for an absorption of the very small UV fraction of the terrestrial solar spectrum. Clearly, photocatalysts adsorbing in the visible region would be advantageous, and therefore, iron oxide photocatalysts attract a steadily growing interest. Their absorption near the maximum of the solar spectrum (bandgap α-/γ-Fe_2_O_3_ ≈ 2.2 eV (Litter and Blesa 1988)), cost-efficiency, abundancy, and non-toxicity makes them promising candidates as photocatalyst in environmental waste management (Khedr et al. 2009; Maji et al. 2012; Wu et al. 2015).

**Fig. 1:**
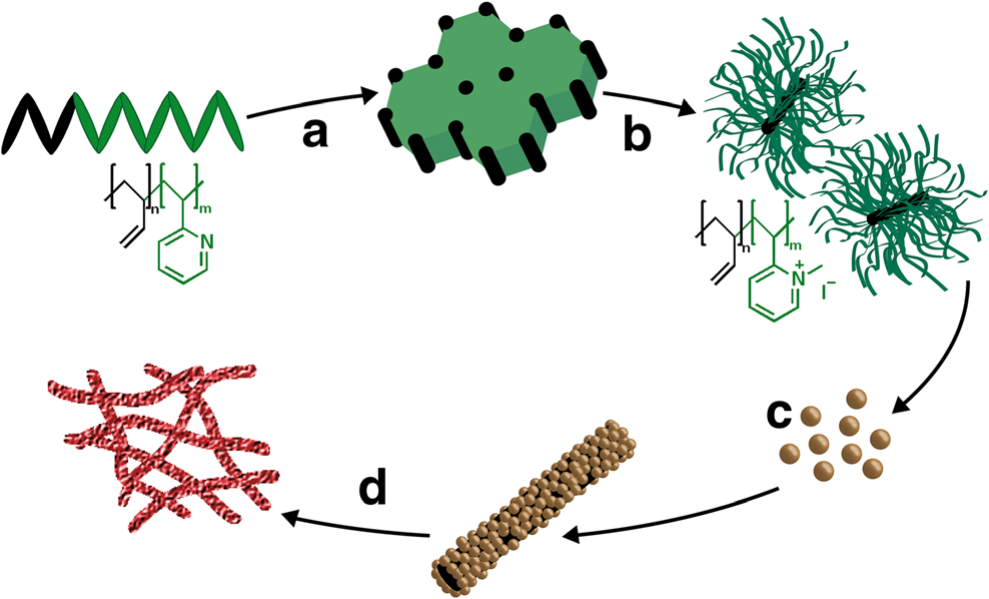
General scheme for catalyst preparation: hexagonally packed polybutadiene (PB) cylinders embedded in a poly(2-vinylpyridine) (P2VP) matrix resulting from the self-assembly of the diblock copolymer (A). Crosslinking of the PB cylinders and quaternization of the matrix yields water-soluble cationic CPBs (B) which can be used as template that is loaded with Fe_2_O_3_ nanoparticles (C). Calcination transforms the hybrid material into a mesostructured non-woven consisting of pure Fe_2_O_3_ (D)

In this work, Fe_2_O_3_ was mesostructured by electrostatic self-assembly of citrate-stabilized Fe_2_O_3_ nanoparticles with cationic cylindrical polymer brushes (CPBs) acting as structure-directing agent (SDA; Fig. 1). After template removal by calcination in air, nanotubes were obtained that are aggregated into non-woven structures, which resemble the structure of the previously published mesostructured ZnO. The mesostructured Fe_2_O_3_ was tested in the photodegradation of ciprofloxacin under irradiation with the terrestrial solar spectrum and compared to commercially available Aeroxide® TiO_2_ P25 and mesostructured ZnO.

## Materials and methods

### Chemicals

If not mentioned otherwise, all chemicals were purchased from Sigma-Aldrich, Germany, and used as-received without further purification.

### Synthesis of the diblock copolymer template

The polybutadiene-*block*-poly(2-vinylpyridine) (BV) diblock copolymer was synthesized by sequential living anionic polymerization in THF initiated with *sec*-butyllithium as published (Yelamanchili et al. 2009; Müllner et al. 2012; Bojer et al. 2017). The determination of composition and molecular weight (by a combination of ^1^H-NMR spectroscopy (Bruker Ultrashield AC300) and matrix-assisted laser desorption-ionization–time of flight mass spectrometry (MALDI-TOF MS, Bruker Reflex III)) resulted in B_19_V_81_^60^. In this notation, the subscripts denote the mass fraction of the corresponding block in wt-% and the superscript gives the number-average molecular weight in kg mol^−1^. The molar mass dispersity of Ð_M_ = 1.02 was determined by size-exclusion chromatography in THF.

A hexagonally ordered film of typically 1 g B_19_V_81_^60^ with 0.1 g photoinitiator (Lucirin-TPO, BASF) was prepared by solvent-casting from chloroform and was subsequently cross-linked using a UV hand lamp (Hoenle, 2 h per side). Afterward, the film was dispersed in 200 mL chloroform and the P2VP block was quaternized by dropwise adding of 3 mL iodomethane (99 %). Once the solvent and iodomethane solution were evaporated, the quaternized product was washed with pentane, dissolved in MilliQ water, and lyophilized.

### Synthesis of the iron oxide nanoparticle dispersion

Iron oxide nanoparticles were synthesized as follows: iron(III) chloride hexahydrate (21.62 g, 80 mmol) and iron(II) chloride tetrahydrate (7.96 g, 40 mmol) were dissolved in 50 mL and 16 mL MilliQ water, respectively. The iron(II) solution was acidified with 2 mL hydrochloric acid (25 %). Both solutions were mixed under vigorous stirring and added slowly to 650 mL ammonia solution (15 %). The black dispersion obtained was heated to 60 °C for 10 min to evaporate excess of ammonia. After sedimentation with a magnet, the residue was washed several times with MilliQ water until a pH value of 8 was reached. Nitric acid (2 M, 160 mL) was added for oxidation, and the solution was kept at 80 °C for 5 min. After addition of iron(III) nitrate nonahydrate solution (160 mL, 0.56 M, preheated to 80 °C in order to avoid significant temperature loss), the mixture was stirred for another 25 min at 80 °C. The reddish brown particles were sedimented with a magnet, and the supernatant liquid was discarded. The residue was redispersed in a small amount of MilliQ water, dialyzed for 7 days against sodium citrate tribasic dihydrate (0.005 M, daily exchange with fresh solution) and centrifuged (2 h at 5000 rpm). The nanoparticle dispersion was diluted with MilliQ water to a solid content of 7.5 mg mL^−1^.

### Synthesis of the iron oxide nanotubes

For the synthesis of the Fe_2_O_3_ hybrid material, 150 mg of quaternized B_19_V_81_^60^ was dispersed in 150 mL deionized water followed by addition of 120 mL of the citrate-stabilized nanoparticle dispersion (7.5 g L^−1^) and stirring for 1 day. The brownish precipitate was removed from the supernatant liquid by centrifugation (3 min, 3500 rpm), washed 5 times with MilliQ water and lyophilized. The organic template was removed in a tube furnace at 500 °C for 5 min (180 K h^−1^ heating and cooling rate) under a constant stream of synthetic air (mixture of 21 vol.-% oxygen and 79 vol.-% nitrogen). Sintering leads to the formation of an all inorganic non-woven structure.

### Photocatalytic degradation

The photocatalytic degradation of the anionic antibiotic ciprofloxacin was done in a self-constructed continuous flow reactor (Fig. S1).

The synthesized iron oxide photocatalyst (20 mg) was drop-casted on a glass microfiber filter (Whatman, 37 mm diameter) and 400 mL ciprofloxacin solution (*c* = 2 × 10^−5^ mol L^−1^) with a pH value of 8 (borate/hydrochloric acid buffer) were circulated at a flowrate of 12 L h^−1^. After equilibration, the catalyst was irradiated with the solar spectrum (300-W Xenon arc lamp from LOT Quantum Design, AM1.5 filter, 1000 W m^−2^), and the degradation was monitored using UV-Vis spectroscopy (samples were taken every 5–15 min). Baseline-corrected spectra were recorded in the range of 350–200 nm. The area of the most intense band (240–300 nm) was used for monitoring the degradation with time (Fig. S2).

### Instruments

The distribution of hydrodynamic diameters of the nanoparticles was analyzed with a Particle Analyzer Litesizer 500 (Anton Paar, Germany) at 20 °C in automatic mode. For calculating the number-weighted hydrodynamic particle size distribution, the absorption coefficient and refractive index of Fe_2_O_3_ were used (Polyanskiy 2019). Furthermore, the zeta potential was measured at 20 °C and computed applying the Smoluchowski approximation.

Mössbauer measurements were performed in transmission geometry with a spectrometer operating with a sinusoidal velocity waveform. The source was about 25 mCi of ^57^Co in Rh. The gamma rays were detected with a proportional counter filled with a Kr/CO_2_ mixture. Measurements at liquid He temperature were performed in a liquid He bath cryostat, in which both the source and the absorber were cooled to 4.2 K. The spectra were fitted with appropriate superpositions of Lorentzian lines grouped into quadrupole doublets or magnetic sextets.

Transmission electron microscopy (TEM) measurements of the nanoparticles were performed with a JEOL JEM-2200FS field emission energy filtering transmission electron microscope (FE-EFTEM) operated at an acceleration voltage of 200 kV. Zero-loss filtered micrographs (ΔE ~ 0 eV) were recorded with a bottom-mounted CMOS camera system (OneView, Gatan) and processed with DM 3.3 image processing software (Gatan). Images of the hybrid material were taken on a Zeiss LEO EM992 Omega EFTEM at an acceleration voltage of 200 kV. Zero-loss filtered micrographs (ΔE ~ 0 eV) were recorded with a bottom-mounted CCD camera system (Ultrascan 1000, Gatan) and processed with DM 1.9 image processing software (Gatan). Samples were prepared by drop-coating dilute dispersions onto carbon-coated copper grids and evaporation of the liquid.

Scanning electron microscopy (SEM) images were acquired with a LEO 1530 FESEM equipped with a field emission cathode. Samples were prepared by drop-coating dilute aqueous dispersions onto silicon wafers.

Nitrogen physisorption data were collected with a Quantachrome Autosorb 1 at 77 K after degassing the samples at 383 K for 16 h.

UV-Vis absorption spectra were measured on a Varian Cary 300 using Helma precision cells made of Quartz SUPRASIL (type 100-QS, light path 10 mm). Reflectance spectra of the powdered samples were measured using the Diffuse Reflectance Accessory of a Cary 5000 UV-Vis-NIR Spectrophotometer.

Thermogravimetric analysis (TGA) was performed on a Netzsch TG 209F1 Libra under synthetic air. All samples were measured from 25 to 800 °C at a heating rate of 10 K min^−1^.

Powder X-ray diffraction (PXRD) measurements were done on a STOE Stadi P diffractometer in Debye-Scherrer geometry with monochromatic Ag K_α1_ radiation and a MYTHEN2 4K detector (4 MYTHEN2 R 1K Si-strip detector modules) in moving mode from 2.5 °2Θ–70° 2Θ (Thomae et al. 2019). Rietveld refinement was done with TOPAS Academic applying a fundamental parameters approach for profile fitting. Implemented macros were used to obtain crystallite sizes. The volume-weighted average crystallite size was obtained assuming spherical particles.

Mott-Schottky plots were measured in 0.1 M Na_2_SO_4_ solution (pH = 5.3) and performed on a Zahner Zennium potentiostat. A three-electrode setup with a working electrode of the obtained iron oxide catalyst, a Ag/AgCl (3 M NaCl) reference electrode, and a Pt counter electrode was used. Measurements were conducted from − 0.4 to 1.2 V_RHE_ at 100 Hz.

The PZC was determined using a ParticleMetrix StabiSizer PMX 200C. Thirty milligrams of catalyst in 10 mL MilliQ water were titrated with 0.01 M NaOH while monitoring the streaming potential vs. pH value.

## Results and discussion

### Synthesis of Fe_3_O_3_ nanoparticles

**Fig. 2:**
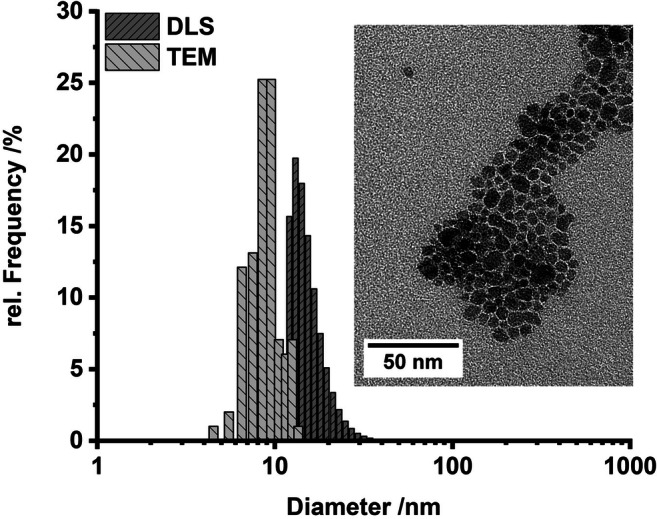
Particle size distribution of γ-Fe_2_O_3_ nanoparticles as determined by DLS measurements (dark gray) and by TEM image analysis (light gray), Inset: TEM image of γ-Fe_2_O_3_ nanoparticles

Particle size distributions of the iron oxide nanoparticles were determined by dynamic light scattering (DLS) and TEM (Fig. 2). DLS gave a mean hydrodynamic particle diameter of 27 nm corresponding to an average number-weighted diameter of gyration of 12 nm (hard spheres (Clara-Rahola et al. 2012); number-weighted hydrodynamic diameter of 16 nm). Furthermore, no large aggregates or agglomerates were observed. Evaluation of 100 nanoparticles from TEM micrographs yielded a median particle diameter of 9 nm. As expected, TEM gives slightly smaller values than the hydrodynamic diameters determined by DLS.

**Fig. 3:**
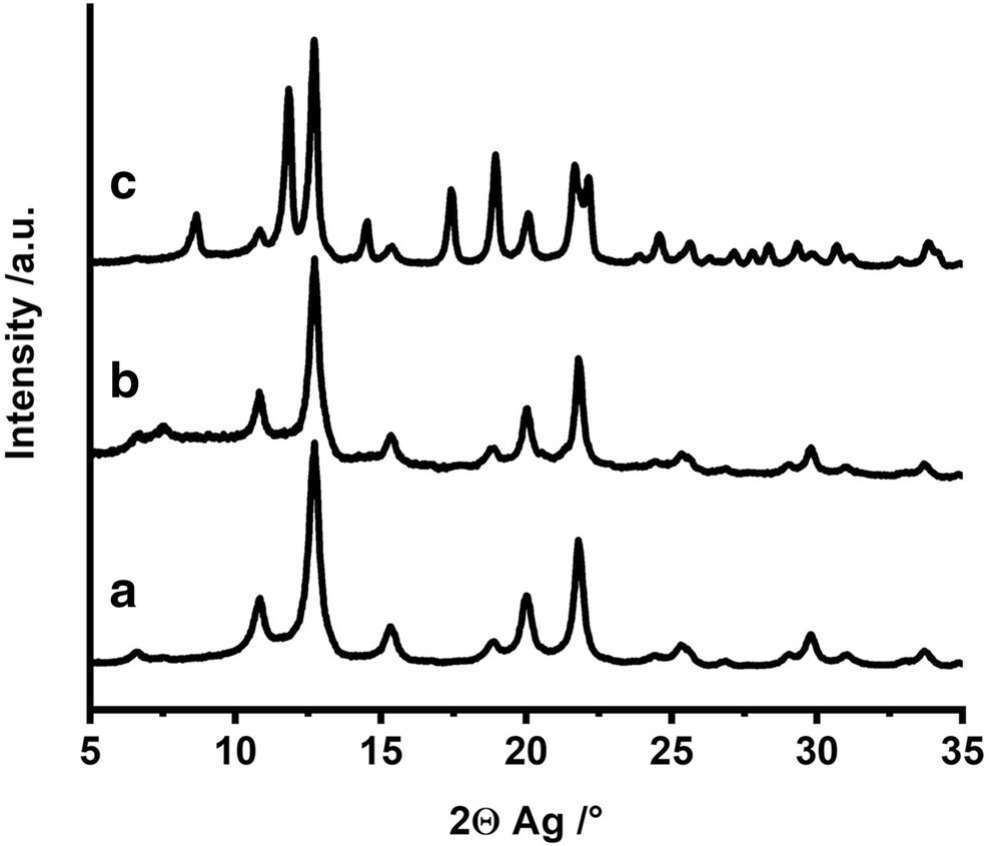
PXRD pattern of (a) γ-Fe_2_O_3_ nanoparticles, (b) hybrid material, and (c) mesostructured Fe_2_O_3_ obtained by calcination

Phase and purity of the iron oxide nanoparticles were discerned via PXRD and Mössbauer spectroscopy. The PXRD pattern of the nanoparticles (Fig. 3a) showed reflexes of γ-Fe_2_O_3_ with broad full width at half maximum as expected for nanoparticles. Rietveld refinement applying TOPAS Academic software (Fig. S3, Table S1) gave average coherently scattering domain sizes of 4.67(3)–6.51(4) nm.

**Fig. 4:**
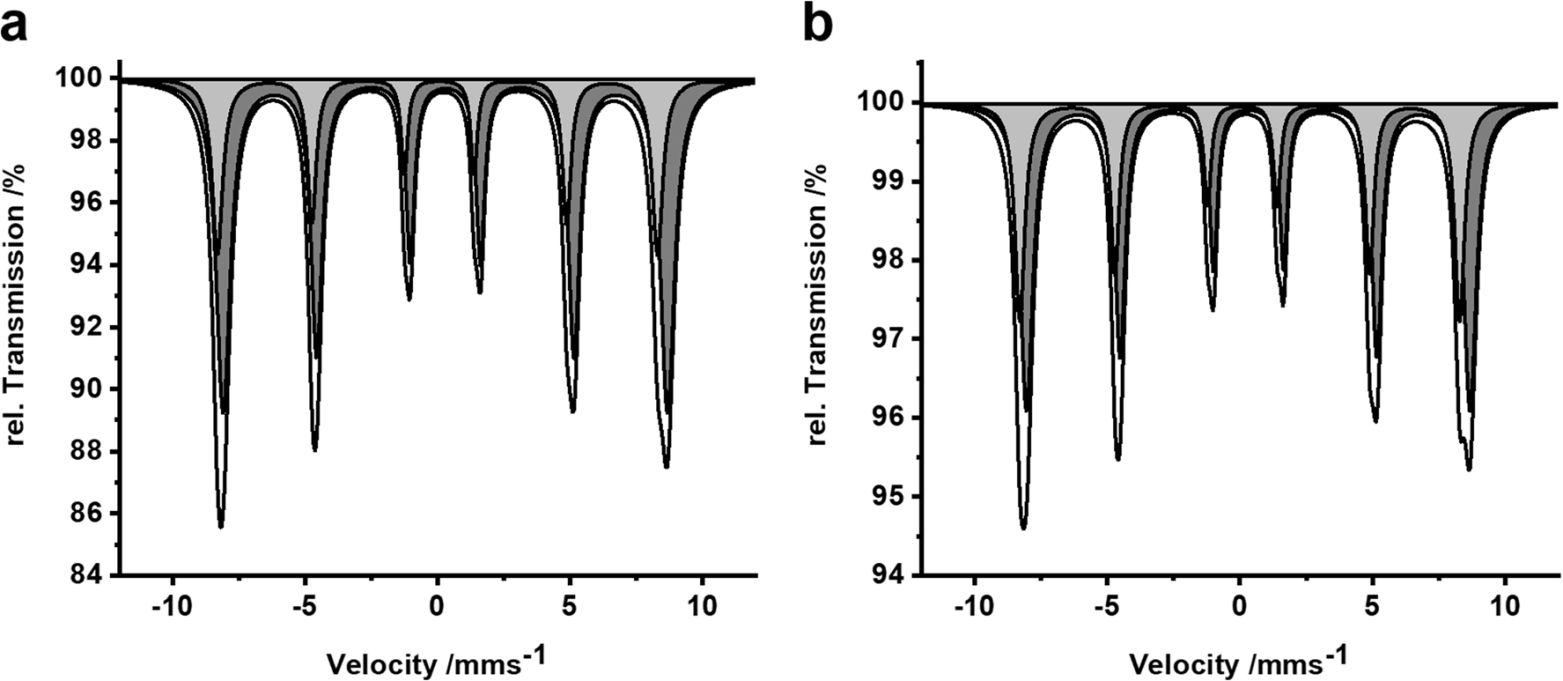
**a** Mössbauer spectrum of the γ-Fe_2_O_3_ nanoparticles (black line) at 4.2 K with fitted sub-spectra (light gray: sextet with δ = 0.05 mm s^−1^; dark gray: sextet with δ = 0.29 mm s^−1^). **b** Mössbauer spectrum of the hybrid material (black line) at 4.2 K with fitted sub-spectra (light gray: sextet with δ = 0.04 mm s^−1^; dark gray: sextet with δ = 0.32 mm s^−1^)

The Mössbauer spectrum recorded at 4.2 K (Fig. 4a) showed a well-developed sextet from magnetic splitting and can be fitted with two sextet subspectra as typically found in γ-Fe_2_O_3_. Isomeric shifts of δ = 0.05 mm s^−1^ and δ = 0.29 mm s^−1^ with hyperfine fields of 52 T and very low quadrupole shifts match with Fe^3+^ atoms in tetrahedral and octahedral sites of γ-Fe_2_O_3_, respectively. No impurities of Fe_3_O_4_ were found. The room temperature Mössbauer spectra (Fig. S4) are typical for γ-Fe_2_O_3_ nanoparticles that undergo superparamagnetic relaxation in a regime where the relaxation is too fast to yield sextet patterns with narrow lines as observed at 4.2 K, but not fast enough to wipe out any magnetic hyperfine interactions, which would result in a quadrupole doublet with narrow lines.

### Formation of the cylindrical hybrid material

**Fig. 5:**
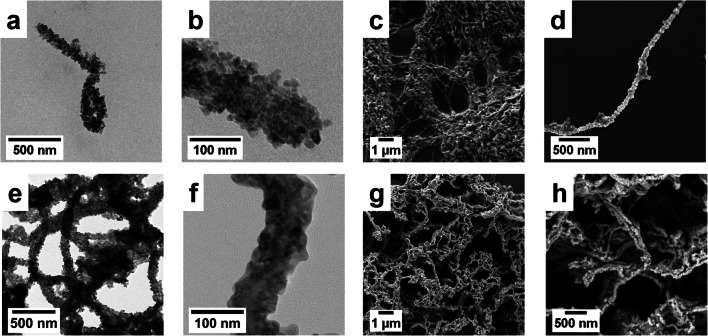
TEM (**a**, **b**) and SEM micrographs (**c**, **d**) of the γ-Fe_2_O_3_ hybrid material; TEM (**e**, **f**) and SEM (**g**, **h**) micrographs of the mesostructured Fe_2_O_3_ obtained after template removal

The citrate-stabilized γ-Fe_2_O_3_ nanoparticles carry a negative surface potential at pH 6 as confirmed by a zeta potential of − 42 mV (Fig. S5). This allowed for a robust hybrid formation via electrostatic interactions with the positively charged CPBs. After removing excess nanoparticles and drying the hybrid material, nanorods (Fig. 5a–d) with a high aspect ratio forming a non-woven microstructure were obtained. The rods were several micrometers in length with a diameter of 118 ± 8 nm as evidenced by grayscale analysis (Fig. S6).

Neither the phase nor the crystallite size of γ-Fe_2_O_3_ is affected by mesostructuring as indicated by Mössbauer and PXRD measurements. Mössbauer spectra recorded at 4.2 K (Fig. 4b) still could be fitted with two sextet sub-spectra similar to the pure nanoparticles. There are no significant changes for isomeric shifts and hyperfine fields (Fe^3+^ in tetrahedral sites: δ = 0.04 mm s^−1^; Fe^3+^ in octahedral sites: δ = 0.32 mm s^−1^) compared to the pure nanoparticles discussed above. In the room temperature spectrum (Fig. S4) a larger contribution of the broad pattern still a sextet shape is observed compared to the as-prepared nanoparticles. This indicates slower relaxation for the nanoparticles attached to the polymer, probably because of magnetic interactions between the adsorbed nanoparticles, which slows down the relaxation.

The amorphous CPBs do not contribute any reflex to the diffraction pattern (Fig. 3b). Coherently scattering domain sizes as obtained by Rietveld analysis (Fig. S8, Table S2) increases only insignificantly to 5.01(4)–7.01(6) nm upon hybrid formation.

**Fig. 6:**
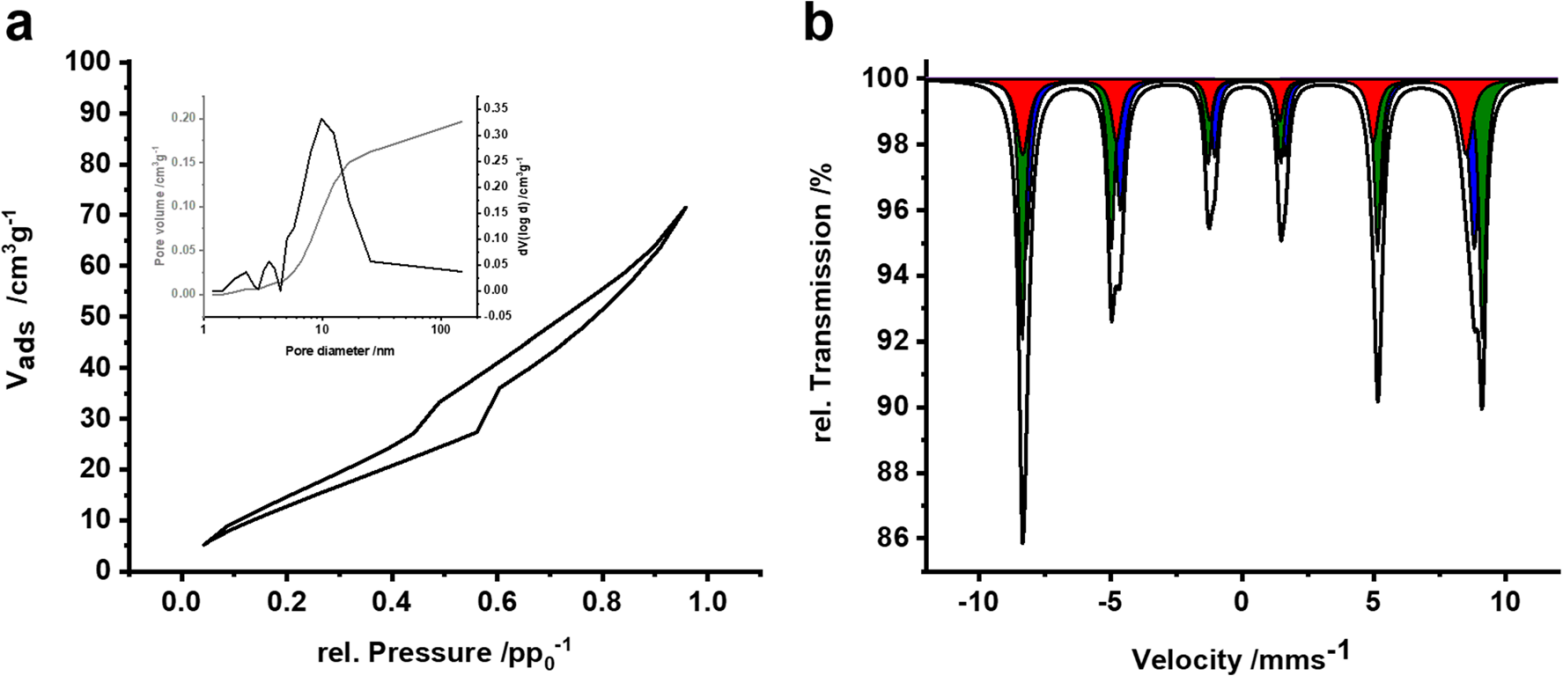
a N_2_-sorption isotherms of the mesostructured Fe_2_O_3_ obtained after template removal. The pore size distribution integrated pore volume as calculated applying the BJH model is shown in the inset. **b** Mössbauer spectrum of the calcined Fe_2_O_3_ nanotubes recorded at 4.2 K (black) with fitted sextet subspectra for maghemite (red: δ = 0.09 mm s^−1^ for Fe^3+^ in tetrahedral sites; blue: δ = 0.3 mm s^−1^ for Fe^3+^ in octahedral sites) and hematite (green: δ = 0.23 mm s^−1^)

The specific surface area of the dried hybrid material as determined from nitrogen physisorption measurements via the Brunauer-Emmett-Teller method (BET) was 64 m^2^ g^−1^ (Fig. 6a). Thermogravimetric analysis results in an iron oxide to CPB ratio of 3:1.

### Pyrolysis to Fe_2_O_3_ nanotubes

To remove the structure-directing CPBs, the hybrid material was calcined in synthetic air at 500 °C. Rod-like morphology and high aspect ratio are maintained (Fig. 5e–h). Upon calcination, the hybrid material was converted into nanotubes as evidenced by grayscale analysis (Fig. S7). The BET-surface area decreases about 15 % from 64 to 54 m^2^ g^−1^ due to Ostwald ripening and sintering of the nanoparticles (Fig. 6a). The pore size distribution calculated by the Barret-Joyner-Halenda (BJH) model shows the presence of some mesopores totaling to a pore volume of ≈ 0.16 cm^3^ g^−1^. The *d*_*50*_ pore diameter for the mesoporous region is 9.5 nm (Fig. 6a, inset).

Calcination triggers partial conversion of the metastable γ-Fe_2_O_3_ to α-Fe_2_O_3_. The Mössbauer spectrum recorded at 4.2 K (Fig. 6b) could be fitted with a total of three sextet subspectra. Two sextets with isomeric shifts of δ = 0.09 mm s^−1^ and δ = 0.30 mm s^−1^ with hyperfine fields of 52 T and low quadrupole shifts are assigned to Fe^3+^ atoms in the tetrahedral and octahedral positions of γ-Fe_2_O_3_. The remaining sextet with an isomeric shift of δ = 0.23 mm s^−1^, a quadrupole shift of ∆ = 0.30 mm s^−1^ and a hyperfine field of 54 T belongs to α-Fe_2_O_3_. It implies that α-Fe_2_O_3_ has undergone the Morin transition, after which a quadrupole shift of ∆ = 0.4 mm s^−1^ is expected for well-crystallized α-Fe_2_O_3_. The lower quadrupole shift found in the calcined sample can be explained by an imperfect spin orientation along the crystallographic *c*-axis. The amount of hematite determined from the 4.2 K spectrum was 40 %, while 51 % results from evaluation of the spectra at room temperature (Fig. S4). The presence of two different iron oxide phases is also confirmed by additional reflexes observed in the PXRD pattern (Fig. 3c). Quantitative Rietveld refinement (Fig. S9, Table S3) yielded coherently scattering domain sizes of 12.17(9)–17.01(11) nm for α-Fe_2_O_3_ and 6.65(9)–9.30(12) nm for γ-Fe_2_O_3_, respectively.

**Fig. 7:**
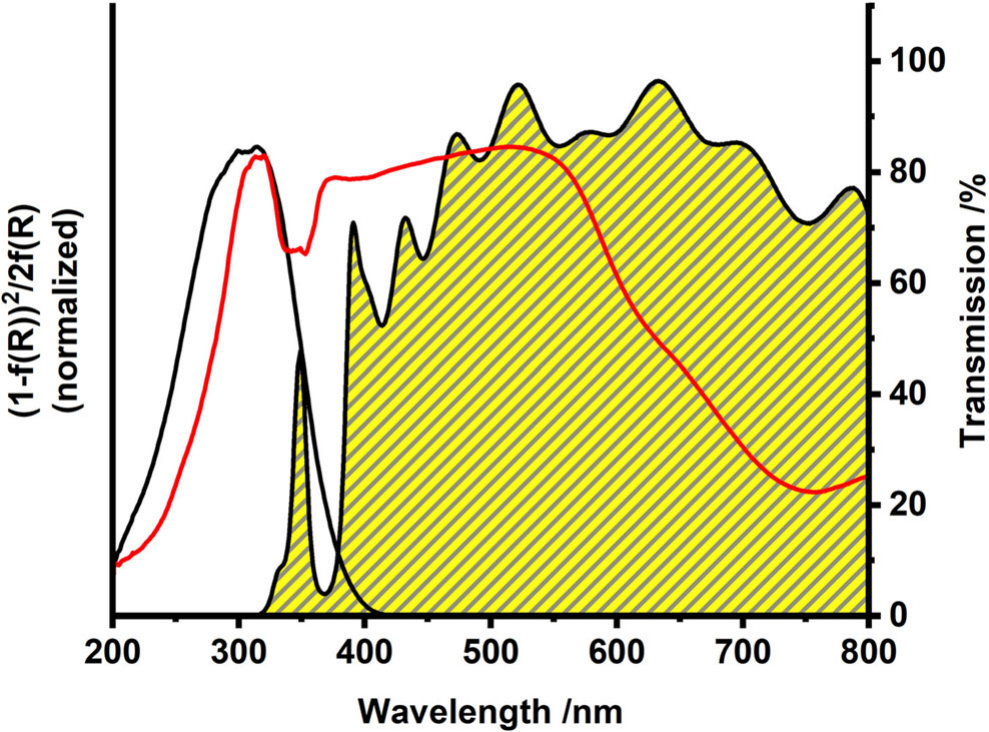
Absorption spectra of Degussa P25 (black) and α/γ-Fe_2_O_3_ nanotubes (red) obtained from diffuse reflection data. The yellow shaded curve represents the transmission spectrum of the AM1.5 filter used in the photocatalytic tests

The calcined Fe_2_O_3_ nanotubes showed a significant higher absorption in the visible range compared to Aeroxide® TiO_2_ P25 (Fig. 7). Using the Tauc plot for diffuse reflection data, the bandgap of the material is calculated as 1.9 eV for the Fe_2_O_3_ sample and 3.4 eV for TiO_2_ (Fig. S10).

The point of zero charge (PZC) for the mesostructured calcined α/γ-Fe_2_O_3_ nanotubes was found at pH = 9.5, as determined by pH-dependent streaming potential measurements. This value is in good agreement with data for iron oxide made via synthesis routes comparable to what has been used here (Kosmulski 2016). Mott-Schottky analyses (Fig. S11) of the nanotubes resulted in a flat band potential of + 0.31 V_RHE_. The positive slope indicates n-typical behavior. The determined flat band potential matches well with values found in the literature for α-Fe_2_O_3_ and is therefore ascribed to the conduction band edge of the α-Fe_2_O_3_ fraction (Gilbert et al. 2013; Sivula and van de Krol 2016).

### Photocatalytic degradation of ciprofloxacin

**Fig. 8:**
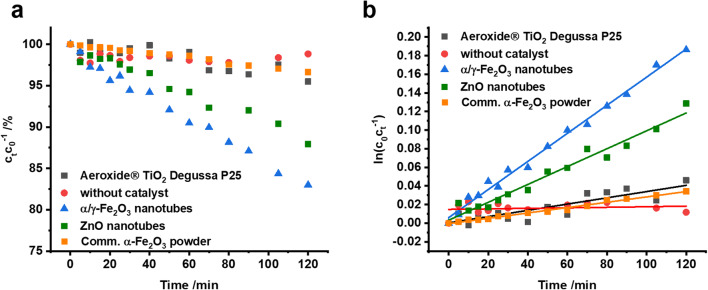
Time-dependent degradation of ciprofloxacin during irradiation with solar spectrum (a) and corresponding pseudo-first-order kinetic plots (b)

The photocatalytic degradation was conducted in a self-constructed continuous flow reactor (Fig. S1). The photocatalytic α/γ-Fe_2_O_3_ nanotubes (20 mg) were placed onto a glass microfiber filter ensuring a homogenous flow. After equilibration in the dark for 120 min, the degradation of ciprofloxacin was monitored for 120 min at pH = 8 applying terrestrial solar radiation. The degradation kinetics were compared to a commercial TiO_2_ photocatalyst (Aeroxide® TiO_2_ P25, 80 % anatase and 20 % rutile with a surface area of approximately 50 m^2^ g^−1^) and ZnO nanotubes studied in a previous work (Bojer et al. 2017). Under the applied conditions, the synthesized α/γ-Fe_2_O_3_ nanotubes are by far superior to both reference materials. After 120 min, the α/γ-Fe_2_O_3_ nanotubes removed 17% of ciprofloxacin whereas only 4.5% and 12% of the initial ciprofloxacin concentration was degraded when Aeroxide® TiO_2_ P25 or ZnO nanotubes were used (Fig. 8a). A blank measurement without photocatalyst showed that irradiation alone lead to only insignificant degradation of ciprofloxacin (≈ 1%).

Assuming pseudo-first-order kinetics (Gaya and Abdullah 2008), the apparent rate constants *k*’ were determined from the slope of the linear fit in the ln(*c*_0_/*c*_*t*_) *vs. t* plot (Fig. 8a). With a rate constant of *k*’ = 1.51 × 10^−3^ min^−1^, the α/γ-Fe_2_O_3_ nanotubes are 4.5 times faster in photodegrading ciprofloxacin as compared to Aeroxide® TiO_2_ P25 (*k*’ = 3.4 × 10^−4^ min^−1^) and 1.6 times faster than ZnO nanotubes (*k*’ = 9.61 × 10^−4^ min^−1^), respectively.

What are possible reasons for the superior performance of the Fe_2_O_3_ nanotubes?

At slightly basic conditions of pH = 8, which are typically encountered with clinical wastewater (Gartiser et al. 1996), ciprofloxacin is present in its anionic form (IEP at pH = 7.4 (Torniainen et al. 1996)). At the same time, α/γ-Fe_2_O_3_ nanotubes with a PZC at pH = 9.5 carry an oppositely charged positive surface charge. Under these boundary conditions set by the application, adsorption is highly favored because it is driven by Coulomb attraction.

Contrary to α/γ-Fe_2_O_3_ nanotubes, the surface of TiO_2_ is negatively charged at basic pH values, and consequently, the adsorption of anionic ciprofloxacin is electrostatically disfavored. Both ZnO nanotubes as well as α/γ-Fe_2_O_3 3_ nanotubes carry a positive surface charge at the pH of clinical wastewater and consequently are superior in the photodegradation of ciprofloxacin over Aeroxide® TiO_2_ P25.

Both ZnO and TiO_2_, however, have large bandgaps only allowing for absorption of the small UV fraction of the terrestrial solar spectrum. Contrary to these previously studied photocatalysts, the bandgap of Fe_2_O_3_ nanotubes (1.9 eV) leads to an absorption close to the maximum of the solar radiation and a much larger fraction of light can be utilized for photoexcitation and charge carrier generation (Fig. 7). While allowing for filtration, the mesoporous non-woven structure at the same time provides good accessibility to a high specific surface area (54 m^2^ g^−1^, Fig. 6) and ensures a high mass transfer through the catalyst material. Together with shortened diffusion pathways for charge carriers by small crystallite sizes, the overall photocatalytic efficiency is significantly improved compared to commercial available hematite powder (microcrystalline; 3.4 % degradation within 120 min and *k*’ = 2.9 × 10^−4^ min^−1^, Fig. 8). At this point, it is rather unclear if a heterojunction due to slightly varying band positions between the two iron oxide polymorphs within the nanotubes is formed. Heterojunction formation would contribute favorably to charge carrier separation and therefore would be expected to improve photocatalytic activity of the α/γ-Fe_2_O_3_ nanotubes as compared to the reference materials (Liu 2012; Aslam et al. 2018).

**Fig. 9:**
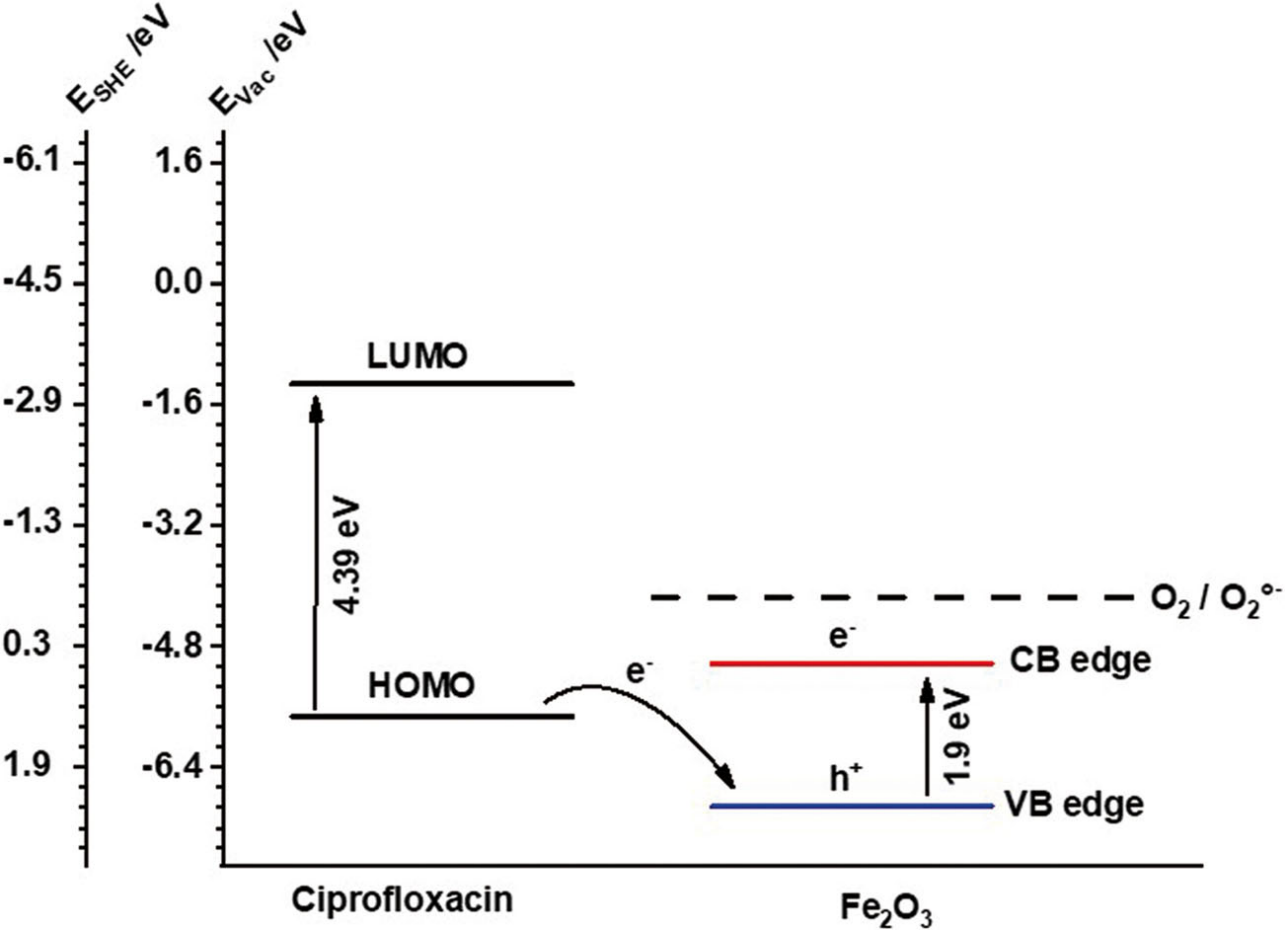
Comparison of the absolute positions of the valence and conduction band of Fe_2_O_3_ nanotubes with HOMO and LUMO levels of ciprofloxacin

The concentration of ciprofloxacin at the surface of the photocatalyst facilitates degradation either by direct reaction with surface trapped charge carriers or intermediate radical species. Comparing the absolute positions of the valence band of Fe_2_O_3_ nanotubes (− 6.9 eV; obtained and converted from diffuse reflection and Mott-Schottky data) with the HOMO level of ciprofloxacin (− 5.72 eV (Stefaniu and Pintilie 2018)), an oxidative degradation is the most likely mechanism (Fig. 9).

Pan et al. (2018) and Mesa et al. (2017) have suggested that methanol and isopropyl alcohol are photooxidized by iron oxide. In this line and consistent with an oxidative mechanism, when adding methanol and isopropyl alcohol as competing hole scavengers, indeed the degradation of ciprofloxacin was diminished to 7 % (*k*’ = 6.1 × 10^−4^ min^−1^) and 5% (*k*’ = 4.1 × 10^−4^ min^−1^), respectively. Despite the high concentration of scavenger applied as compared to the ciprofloxacin concentration, the degradation, however, cannot be suppressed completely. Apparently, the electrostatic interactions between the positively charged photocatalyst surface and anionic ciprofloxacin makes ciprofloxacin competitive with neutral scavengers despite the large difference in concentration. The competitive experiments thus suggest direct oxidation of ciprofloxacin by surface trapped holes to be the dominant degradation mechanism.

The fate of the photoexcited electron in the conduction band and the mechanism for regeneration of the transient reduced α/γ-Fe_2_O_3_ are less obvious. Judging on the published positions of the O_2_/O_2_^−·^ redox potential (− 4.2 eV), reduction of oxygen by electrons in the conduction band of Fe_2_O_3_ nanotubes (− 5.0 eV) should actually be thermodynamically forbidden (Wood 1974). Despite this, applying a method introduced by the group of Ide (Mani et al. 2018), the formation of superoxide radicals could nevertheless be confirmed experimentally. Ide et al. (Mani et al. 2018) showed that the presence of superoxide radicals (O_2_^−·^) can be proven experimentally by the formation of water-insoluble blue formazans on the catalysts surface via reaction of O_2_^−·^ with nitro blue tetrazolium (NBT).

**Fig. 10:**
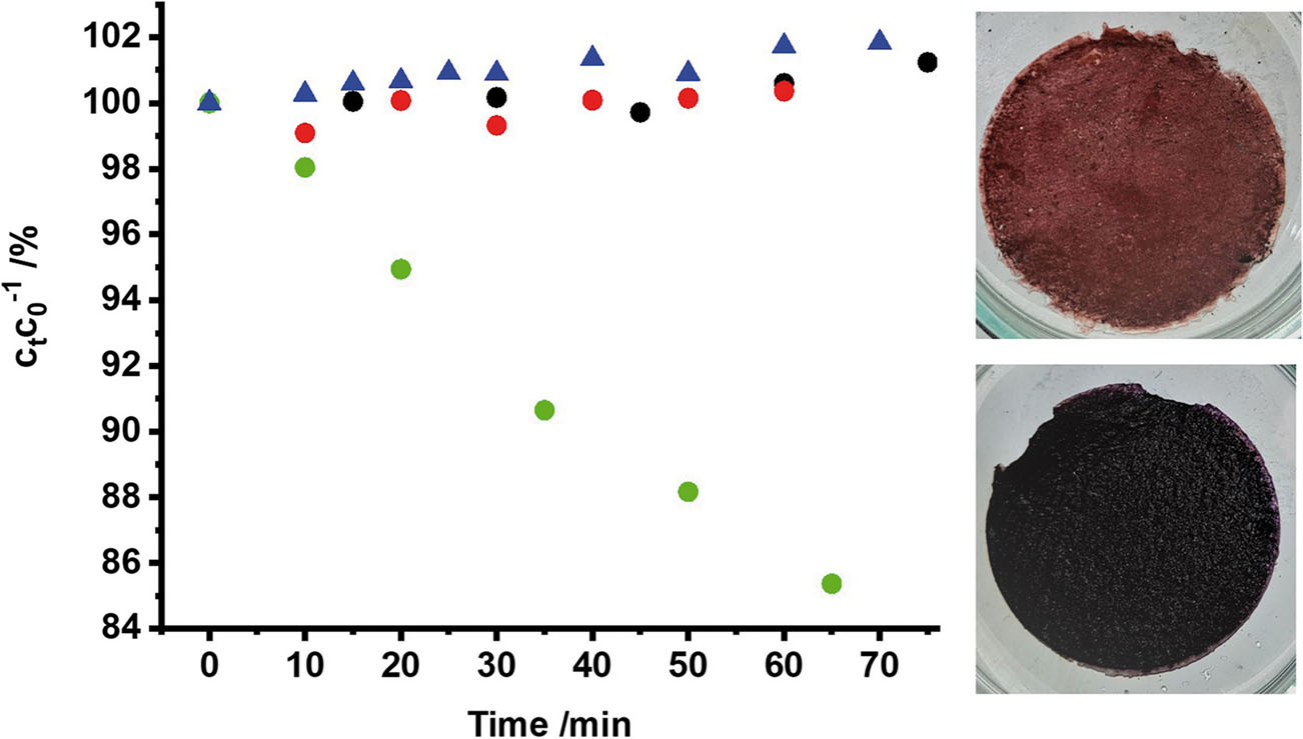
Concentration of nitro blue tetrazolium in the presence of catalyst with (green circles) and without irradiation (black circles) as well as under illumination without catalyst (red circles). Photocatalytic performance of α/γ-Fe_2_O_3_ nanotubes in the degradation of ciprofloxacin in an Ar atmosphere (blue triangles). The pictures on the right side show the photocatalysts color before NBT reduction (top) and the color change due to deposition of water-insoluble formazans (Mani et al. 2018) on the catalyst surface (bottom)

α/γ-Fe_2_O_3_ nanotubes were found to photodegrade 15% of dissolved NBT within an hour when illuminated with terrestrial solar spectrum while blue formazans formed on the surface (Fig. 10). Without light or catalyst, no significant degradation could be observed. A possible explanation for the apparent contradiction might be the recently proposed model by Jaegermann (Lohaus et al. 2018), indicating that the electrochemically measured conduction band minima of iron oxides are most probably polaron trap levels, and the actual conduction band minimum lies much more cathodic, being able to reduce oxygen. Although photoexcited electrons are usually trapped very fast in those polaron trap states, some might still be able to reduce O_2_ during the oxidative degradation of ciprofloxacin.

In summary, our experiments suggest the degradation of ciprofloxacin to involve direct oxidation via holes with parallel reduction of oxygen to form superoxide radicals (Fig. 10). In line with this view, the photodegradation in Ar-saturated solution is significantly less efficient (Fig. 10). Moreover, the superoxide radical intermediate is expected to contribute to the degradation, for instance via disproportionation to H_2_O_2_ (Chong et al. 2010).

Most importantly, the mesostructured catalyst cannot only be applied in a flow-through cell but can also be easily recovered from the fluid reaction medium by filtration. There is no need for expensive techniques like ultracentrifugation.

## Conclusion

Mesostructured Fe_2_O_3_ allows for an efficient photodegradation of ciprofloxacin at the basic pH of clinical wastewaters. Moreover, due to an adsorption band close to the maximum of the solar spectrum, this photocatalyst is superior to the large bandgap semiconductors P25 or ZnO. The smaller bandgap allows to utilize a larger fraction of the solar radiation for charge carrier generation. In addition, the non-woven-like mesostructure minimizes diffusion pathways to the surface while at the same time ensuring a high mass transport through the catalyst as well as simple recycling by filtration.

Scavenger experiments suggest direct oxidation of ciprofloxacin via holes and a reduction of dissolved oxygen gas to form superoxide radicals. Simple O_2_ is thus fully sufficient in this advanced photooxidation process with no need to add expensive H_2_O_2_ triggering a Fenton-type reaction.

## Electronic supplementary material

ESM 1 (DOCX 4209 kb)
