# Supplementary material for: Terrestrial solar radiation driven photodecomposition of ciprofloxacin in clinical wastewater applying mesostructured iron(III) oxide
Source: Environ Sci Pollut Res Int. 2020 Sep 29;28(5):6222–31. doi: 10.1007/s11356-020-10899-6 (PMC7838145; doi:10.1007/s11356-020-10899-6)
Supplement: Supplementary file 1 — (DOCX 4209 kb) [file 11356_2020_10899_MOESM1_ESM.docx]

**Supplementary Information**

**Terrestrial Solar Radiation Driven Photodecomposition of Ciprofloxacin in Clinical Wastewater Applying Mesostructured Iron(III) Oxide**

**Daniel R. Wagner^a^, Kevin Ament^a^, Lina Mayr^a^, Thomas Martin^a^, André Bloesser^a^, Holger Schmalz^a^, Roland Marschall^a^, Friedrich E. Wagner^b^, Josef Breu^a^***

**^a^ Bavarian Polymer Institute (BPI) and Department of Chemistry, University of Bayreuth, 95440 Bayreuth, Germany**

**^b^ Physics-Department E15, Technical University of Munich, James-Franck-Straße, 85748 Garching, Germany**

*Corresponding author. Bavarian Polymer Institute (BPI) and Department of Chemistry, University of Bayreuth, 95440 Bayreuth, Germany

*Email address:* josef.breu@uni-bayreuth.de


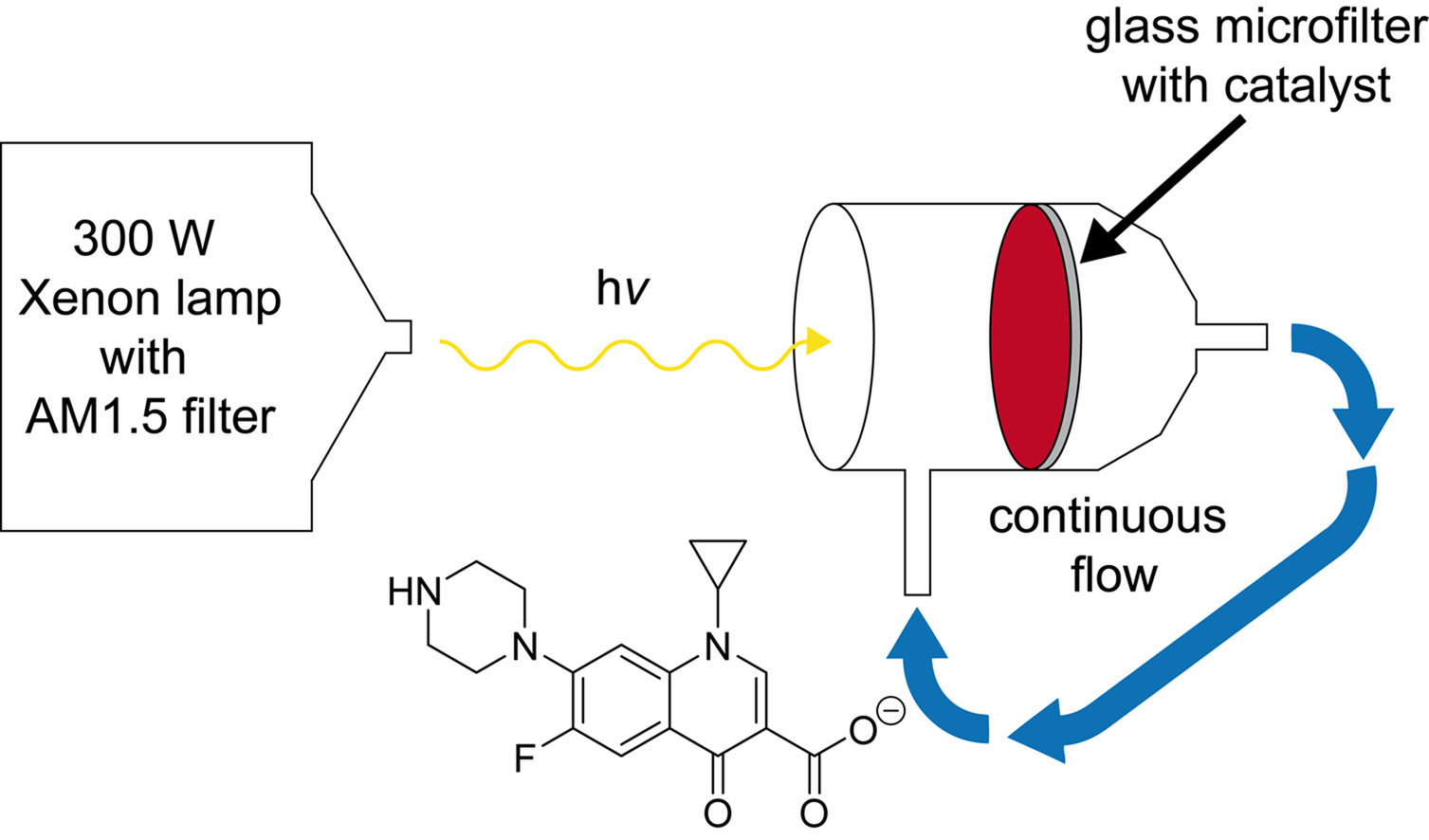


**Fig. S1** Schematic reactor setup


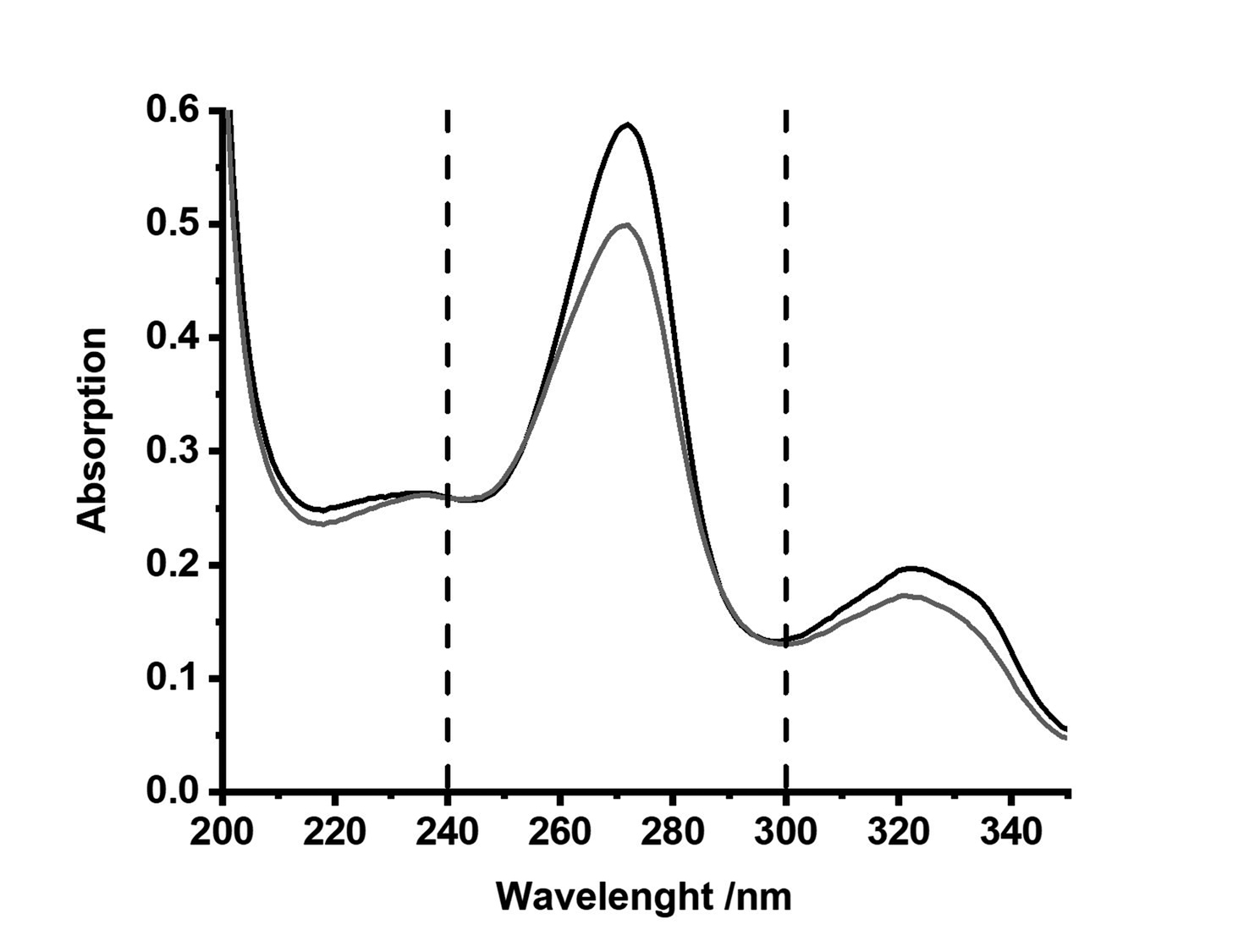


**Fig. S2** UV-Vis absorption spectra of ciprofloxacin at the beginning of the degradation experiment (black) and after 120 min (gray). For calculations the areas of the band between 240 nm and 300 nm (vertical dashed lines) were used


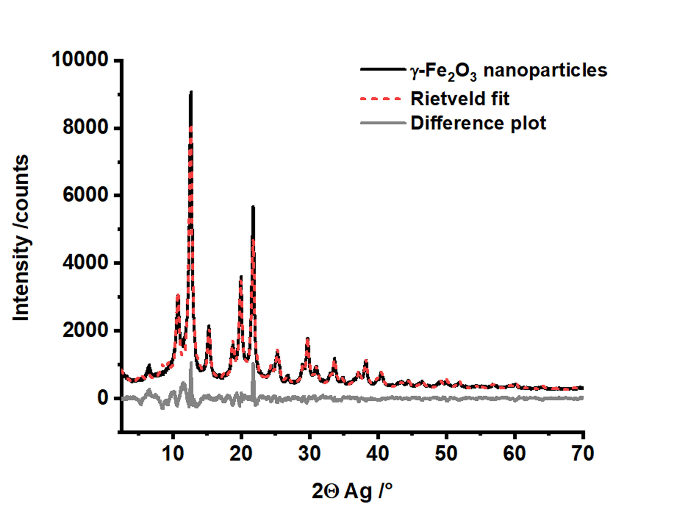


**Fig. S3** PXRD data of the γ-Fe_2_O_3_ nanoparticles (black), calculated Rietveld refinement (red; CIF: 87119-ICSD) and difference plot (dark grey)

**Table S1** Refinement parameters for γ-Fe_2_O_3_ nanoparticles

|  |  |
| --- | --- |
| **Space group** | P4332 |
| **R (exp)** | 3.65 |
| **R(p)** | 6.29 |
| **R(wp)** | 8.49 |
| **R(bragg)** | 2.82 |
|  |  |
| **Cell constants [Å]** |  |
| **a** | 8.353(1) |
| **b** | 8.353(1) |
| **c** | 8.353(1) |
| **α** | 90 |
| **β** | 90 |
| **γ** | 90 |
|  |  |
| **Zero point correction** | 0.051(3) |
|  |  |
|  |  |


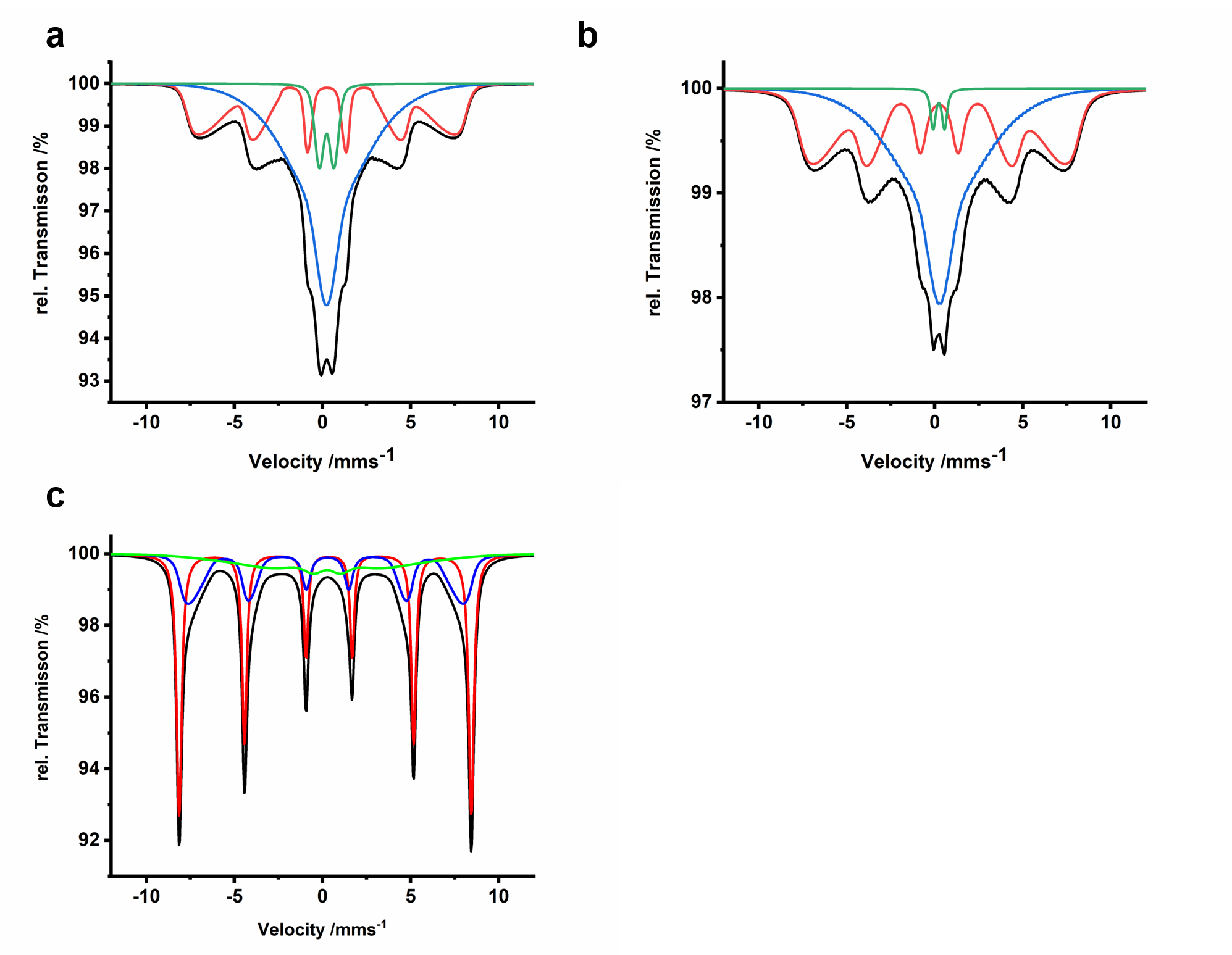


**Fig. S4** Room temperature Mössbauer spectra of the as-prepared γ-Fe_2_O_3_ nanoparticles (a), of the hybrid material (b) and of the calcined Fe_2_O_3_ nanotubes (c). Even though the spectral shapes of the nanoparticles and hybrid material are due to dynamic relaxation effects, they were fitted with a superposition of static patterns. A reasonably good fit was obtained with a sextet with broad lines approximated by a pattern corresponding to a gaussian distribution of hyperfine fields around a mean value of 47 T, by a second broad distribution of hyperfine fields with a small mean field of about 10 T representing a collapsed sextet, and a quadrupole doublet. These three components may be interpreted as being due to a distribution of different relaxation rates probably resulting mainly from the distribution of particle sizes. The calcined sample shows the narrow sextet of α-Fe_2_O_3_ with a fractional area of 51 %. The remainder is a broad sextet of γ-Fe_2_O_3_ approximated by two broad sextet patterns with better defined magnetic splitting


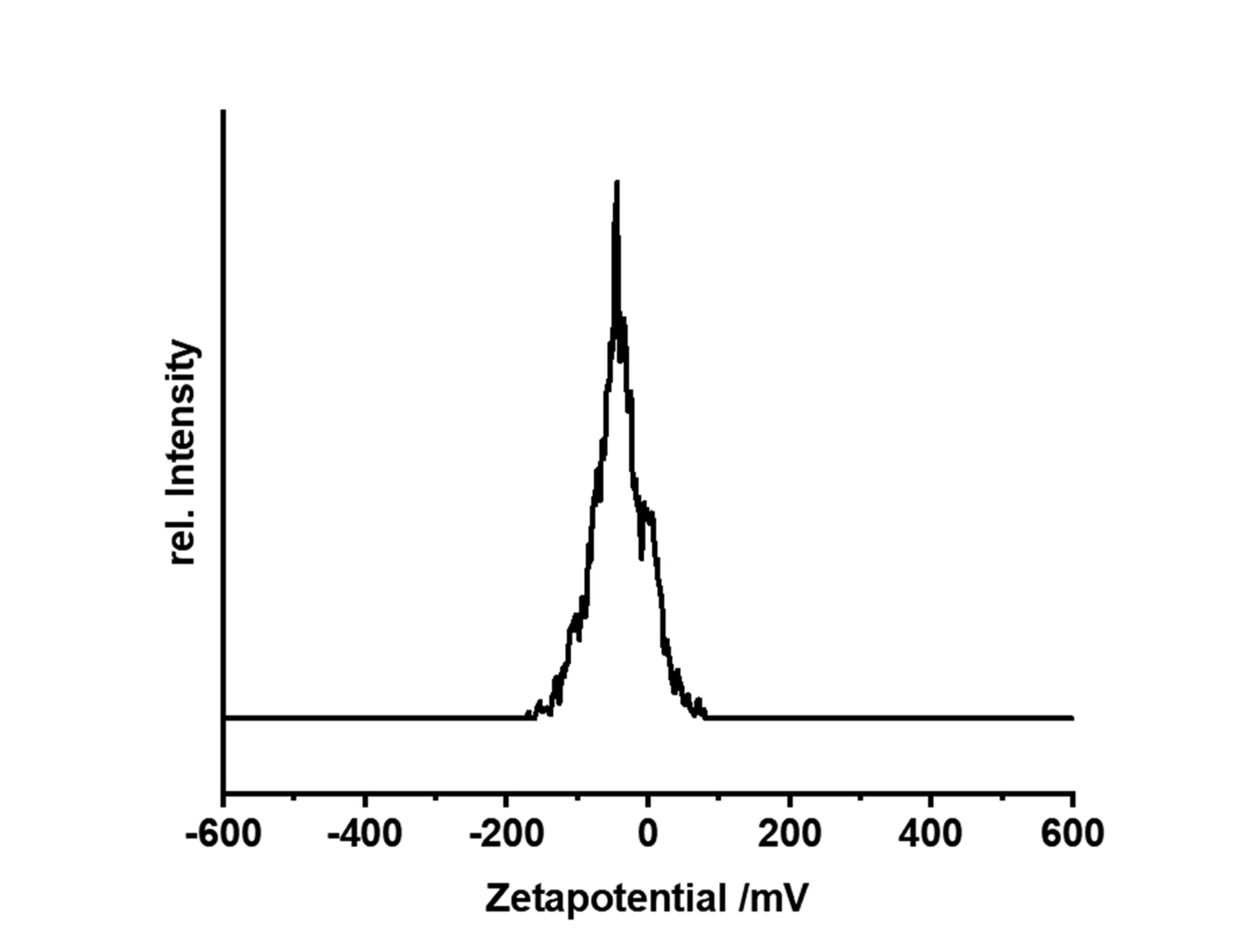


**Fig. S5** Zeta potential of citrate-stabilized γ-Fe_2_O_3_ nanoparticles


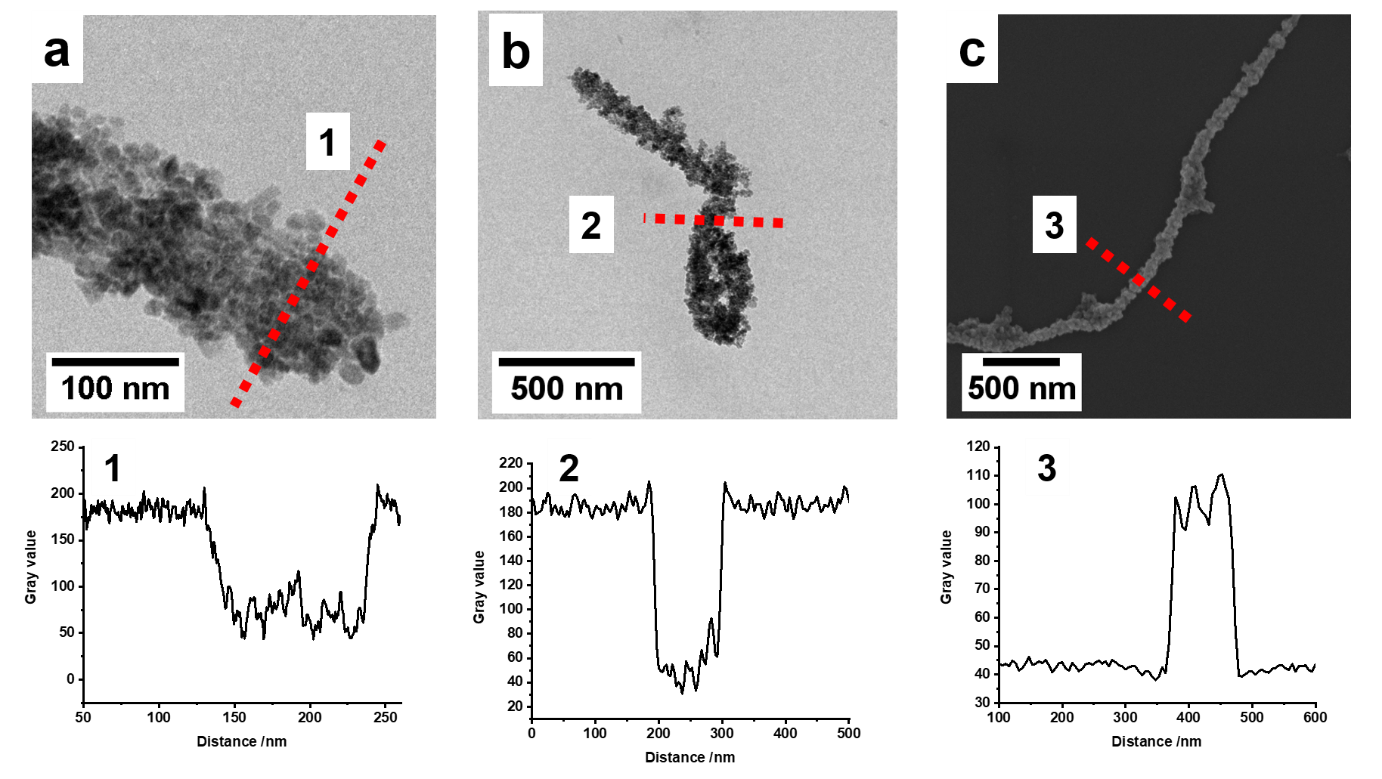


**Fig. S6** Gray scale analysis of the hybrid material (a+b: TEM; c: SEM)


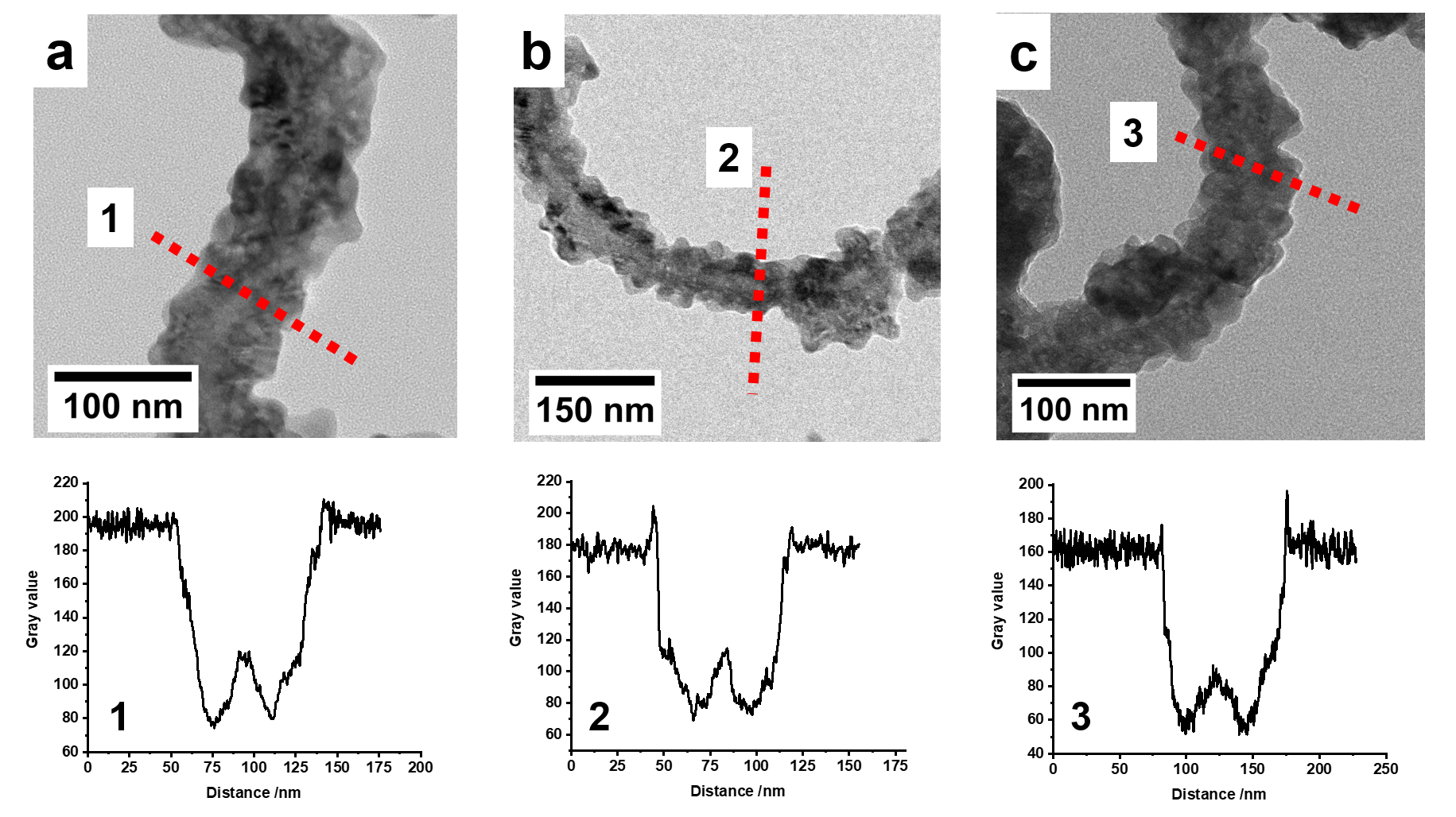


**Fig. S7** Gray scale analysis of TEM micrographs after template removal


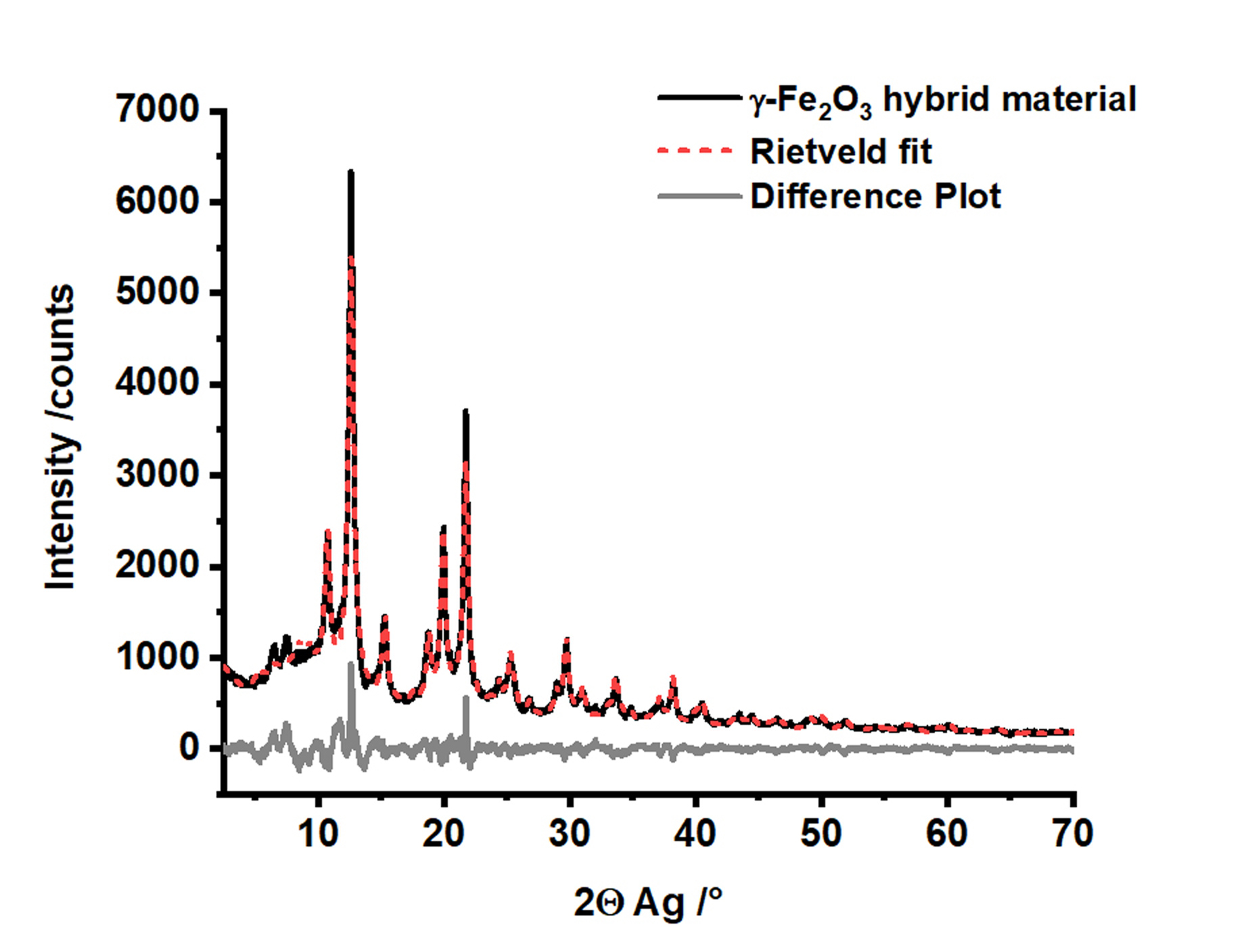


**Fig. S8** PXRD data of the hybrid material (black), calculated Rietveld refinement (red; CIF: 87119-ICSD) and difference plot (dark grey)

**Table S2** Refinement parameters for the hybrid material

|  |  |
| --- | --- |
| **Space group** | P4332 |
| **R (exp)** | 4.11 |
| **R(p)** | 6.29 |
| **R(wp)** | 8.01 |
| **R(bragg)** | 3.20 |
|  |  |
| **Cell constants [Å]** |  |
| **a** | 8.358(1) |
| **b** | 8.358(1) |
| **c** | 8.358(1) |
| **α** | 90 |
| **β** | 90 |
| **γ** | 90 |
|  |  |
| **Zero point correction** | 0.013(3) |


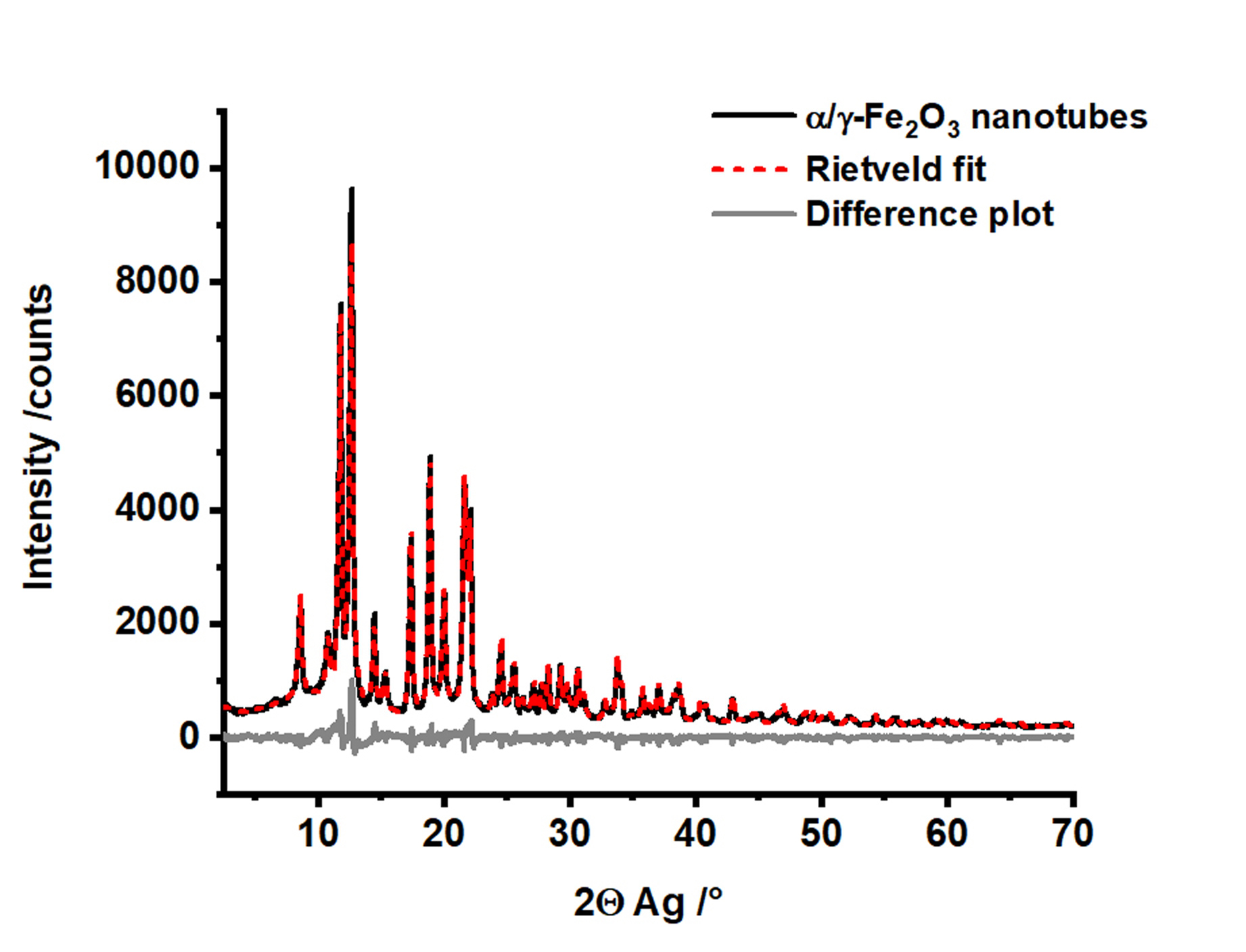


**Fig. S9** PXRD data after template removal (black), calculated Rietveld refinement (red; γ-Fe_2_O_3_ (CIF: 87119-ICSD)+ α-Fe_2_O_3_ (CIF:15840-ICSD)) and difference plot (dark grey)

**Table S3** Refinement parameters for the α/γ-Fe_2_O_3_ nanotubes

|  |  |
| --- | --- |
| **R (exp)** | 3.87 |
| **R(p)** | 5.38 |
| **R(wp)** | 6.70 |
| **Zero point correction** | 0.034(2) |
| **γ-Fe_2_O_3_** | |
| **Space group** | P4332 |
|  |  |
| **Cell constants [Å]** |  |
| **a** | 8.338(1) |
| **b** | 8.338(1) |
| **c** | 8.338(1) |
| **α** | 90 |
| **β** | 90 |
| **γ** | 90 |
|  |  |
| **R(bragg)** | 2.51 |
| **α-Fe_2_O_3_** | |
| **Space group** | R-3cH |
|  |  |
| **Cell constants [Å]** |  |
| **a** | 5.034(2) |
| **b** | 5.034(2) |
| **c** | 13.752(8) |
| **α** | 90 |
| **β** | 90 |
| **γ** | 120 |
|  |  |
| **R(bragg)** | 2.97 |


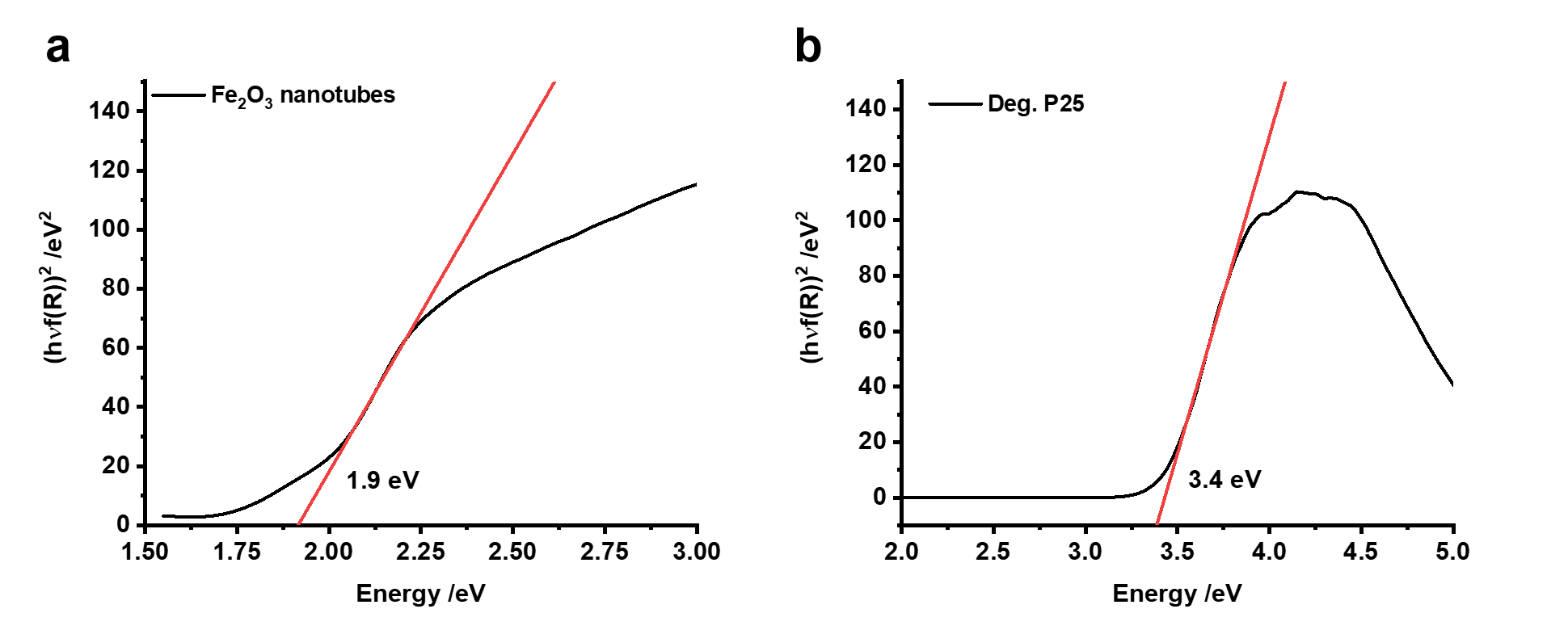


**Fig. S10** Optical band gap estimation for Fe_2_O_3_ nanotubes (a) and Degussa P25 (b) applying the Kubelka-Munk relation for diffuse reflection data


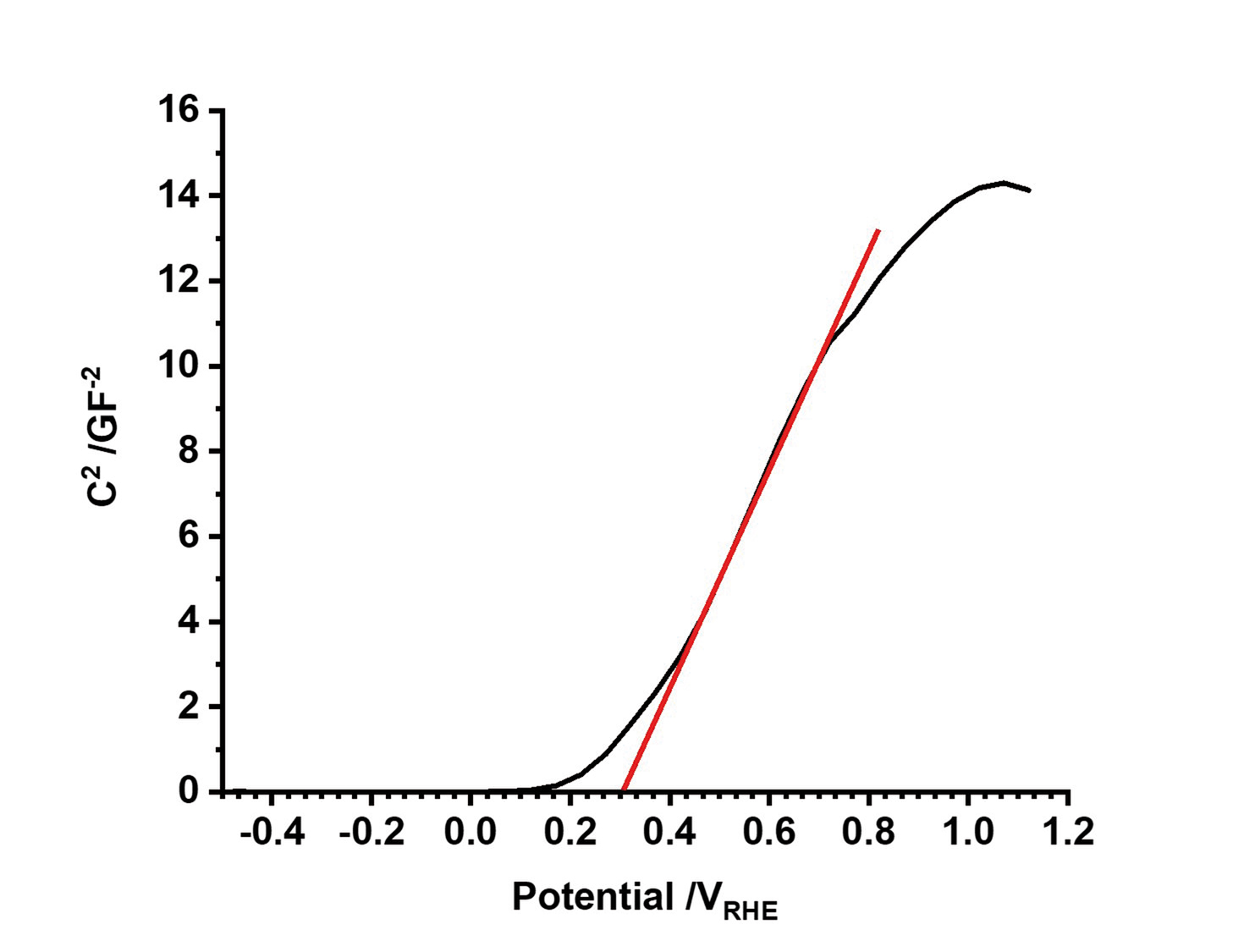


**Fig. S11** Mott-Schottky plot of the calcined Fe_2_O_3_ nanotubes
